# Pessimism, diet, and the ability to improve dietary habits: a three-year follow-up study among middle-aged and older Finnish men and women

**DOI:** 10.1186/s12937-018-0400-8

**Published:** 2018-10-15

**Authors:** Mikko Pänkäläinen, Mikael Fogelholm, Raisa Valve, Olli Kampman, Markku Kauppi, Erja Lappalainen, Jukka Hintikka

**Affiliations:** 1 Department of Psychiatry, Päijät-Häme Central Hospital, Keskussairaalankatu 7, FI-15850 Lahti, Finland; 2 Department of Food and Environmental Sciences, University of Helsinki, Helsinki, Finland; 3 Department of Psychiatry, Seinäjoki Hospital District, Seinäjoki, Finland; 4 School of Medicine, University of Tampere, Tampere, Finland; 5 Department of Internal Medicine, Päijät-Häme Central Hospital, Lahti, Finland

**Keywords:** Pessimism, Optimism, Life orientation test – revised, Dietary habits

## Abstract

**Background:**

Dietary habits have a great influence on physiological health. Even though this fact is generally recognized, people do not eat as healthily as they know they should. The factors that support a healthy diet, on the other hand, are not well known. It is supposed that there is a link between personal traits and dietary habits. Personal traits may also partially explain why some people manage to make healthy dietary changes while some fail to do so or are not able to try to make changes even when they desire to do so. There is some information suggesting that dispositional optimism plays a role in succeeding in improving dietary habits. The aim of this study was to determine the role of optimism and pessimism in the process of dietary changes.

**Methods:**

Dispositional optimism and pessimism were determined using the revised Life Orientation Test in 2815 individuals (aged 52–76 years) participating in the GOAL study in the region of Lahti, Finland. The dietary habits of the study subjects were analysed. After 3 years, the subjects’ dietary habits and their possible improvements were registered. The associations between dispositional optimism and pessimism, dietary habits at baseline, and possible changes in dietary habits during the follow-up were studied with logistic regression. We also studied if the dietary habits or certain lifestyle factors (e.g. physical exercising and smoking) at baseline predicted success in improving the diet.

**Results:**

Pessimism seemed to correlate clearly negatively with the healthiness of the dietary habits at baseline – i.e. the higher the level of pessimism, the unhealthier the diet. Optimism also showed a correlation with dietary habits at baseline, although to a lesser extent. Those who managed to improve their dietary habits during follow-up or regarded their dietary habits as healthy enough even without a change were less pessimistic at baseline than those who failed in their attempts to improve their diet or did not even try, even when they recognized the need for a change.

**Conclusions:**

Pessimistic people are more likely to eat an unhealthy diet than others. Pessimism reduces independently the possibilities to improve dietary patterns.

## Background

Despite the well-known connection between dietary habits and health, many people do not eat what is recommended as a healthy diet [1]. Dietary habits are related e.g. to the risk of coronary heart disease (CHD) [2] and improving dietary habits has showed significant cardioprotective effects in a secondary prevention program among women with CHD [3].

While the intention to prevent diseases is usually thought to be an important reason for a healthier diet, psychosocial and lifestyle related factors seem to be one of the major causes for not eating healthily. The most common factors mentioned in preventing a healthy diet are a lack of time, a reluctance to give up favourite foods, and a lack of motivation and willpower [4–6]. A healthy diet is also thought to be more expensive than unhealthy one, even if this belief seems to be false [7, 8].

The terms optimism and its antonym pessimism derive from Latin words ‘optimus’ and ‘pessimus’, respectively (the first meaning ‘the best’ and the latter meaning ‘the worst’ [9]) and they are used in describing people’s outlook and expectations concerning their future. Persons who have a feeling or belief that good things will happen in the future are called optimists and they are said to see “the glass as half-full rather than half-empty”. Pessimists in turn generally feel that bad things are more likely to happen than good things [10]. Optimism is regarded in psychology as a cognitive, affective and motivational construct [11]. On other words, optimists not only think, but also feel positively about the future. Like other personal trait, also optimism and pessimism develop during the childhood and early adulthood influenced by both heritage and environment [12, 13], and unlike e.g. mood, the construct of optimism (including both optimistic and pessimistic properties) is thought to be quite stable after it has evolved, regardless of negative or positive incidents [14, 15].

People are often categorized as optimists or pessimists. This can lead to the conclusion that optimism and pessimism are the two extremities of the same unidimensional continuum (dispositional optimism). Nevertheless, the concept of optimism itself has long been controversial: there is debate over whether the optimism construct should be seen as one bipolar dimension or if optimism and pessimism should be seen as two separate dimensions that exist simultaneously and may be unattached to each other.

Optimism is sometimes confused with other concepts, e.g. features like the sense of control [16], self-efficacy [17] and hope [18]. There are still differences with these terms. Unlike the concept of optimism, these properties include also how the desired outcomes are expected to happen. For example, a person with high self-efficacy believes that his/her personal efforts or skills are what will determine the positive outcome while an optimist does not rely on his/her own abilities.

Numerous psychosocial factors have been noted to influence dietary behaviour. A connection seems to exist between psychosocial features and current diet, and also between psychosocial features and the ability to improve the diet. Psychosocial features of interest include e.g. socio-economic status, willpower, self-efficacy, and satisfaction with life. There are many studies on the associations between these psychosocial factors and healthy eating [4, 19–24], but the number of studies concerning the optimism construct and dietary habits is quite small. The findings of these few studies suggest that there might be a positive connection between optimism and the willingness and capability to eat in a healthier way [25–29]. In all of these studies on the connection between the optimism construct and dietary habits, optimism has been associated with healthier diet and/or pessimism vice versa. In a study on young Finnish adults, unipolarly measured optimism had an influence on dietary habits, and pessimism was linked to an unhealthy diet [25]. In a study on elderly men, a low level of optimism was associated with an unhealthy lifestyle, including unhealthy dietary habits [29]. In the large Women’s Health Initiative study, high optimism was strongly related to healthier eating habits and greater levels of success in improving dietary habits [26, 27]. In a study on Polish menopausal women, optimism was positively correlated with a healthier diet [28]. However, we did not find any previous studies with general population samples focusing on the dietary habits and the optimism construct that would handle optimism and pessimism as independent factors. We conducted this 3-year follow-up study on middle-aged and older Finnish men and women to determine whether optimism and pessimism are factors that associate with dietary habits and predict success in improving those habits.

## Methods

The GOAL study (Good Ageing in Lahti Region) started in 2002. Its aim was to determine and improve the health and well-being of the ageing population of the region of Lahti, a city in southern Finland. The entire project consisted of a cohort study and several community-based interventions and it lasted for 10 years. In the present study, data from baseline (year 2002) and 3-year follow-up (year 2005) of the cohort study were used.

The cohort study group consisted of a stratified (age, sex, municipality) random sample of men and women born in 1926–30, 1936–40, and 1946–50. The study participants were drawn from the population registry of all 14 municipalities in the Lahti region. A total of 4272 subjects were invited, and 2815 (66%) participated.

At the beginning of the GOAL study, cross-sectional data on the dietary habits, current health, and lifestyles of the study subjects were gathered by using questionnaires. The study subjects were asked about their recent dietary habits with a food frequency questionnaire (FFQ) where different foods were divided into 24 categories. The respondents were asked how often they had consumed the foods in each category during the last 7 days. The answers were scaled from 1 (not at all) to 4 (on 6 or 7 days). Study subjects were measured for height and weight and their body mass indexes (BMI) were calculated. According to their smoking habits, the study subjects were divided into two groups, ‘daily smokers’ (i.e. those who smoked every day, regardless of the amount) and ‘non-daily smokers’. Study subjects who used five or more units of alcohol (one unit = 12 g EtOH) in one sitting formed the ‘heavy drinkers’ group, while the rest were ‘non-heavy drinkers’. The study subjects were asked if they had been diagnosed with CHD by a doctor. Finally, the subgroup ‘regular physical exercise’ was formed to include those who exercised for 30 min at least twice a week. In addition to the questionnaires, several blood tests were taken. The samples were measured for the levels of blood glucose and cholesterol, among other things.

Levels of dispositional optimism and pessimism were measured by using the revised version of the Life Orientation Test (LOT-R). The test was initially developed in the mid-1980s to assess the beneficial effects of optimism on psychological and physiological health (Life Orientation Test (LOT)) [30]. The scale was re-evaluated and revised (LOT-R) later to focus its item content more closely on the subject’s expectations of the future [31].

LOT-R includes six statements, three worded positively for optimism (e.g. ‘In uncertain times, I usually expect the best’) and three worded negatively to indicate pessimism (e.g. ‘If something can go wrong for me, it will’). The respondents are asked to indicate how much they agree with the statements in general, as expressed on a scale from 0 (‘I disagree a lot’) to 4 (‘I agree a lot’). A higher score refers to greater optimism or greater pessimism depending on the statement. Originally, both LOT and LOT-R were thought to be unidimensional scales, but later studies have suggested that they may have two separate independent dimensions, namely optimism and pessimism [32–36]. In the one-dimensional bipolar model with optimism and pessimism as opposites, the optimism scores and pessimism scores are calculated together and they might cancel out and hide each other’s results. Our previous study showed clearly that in this study sample, LOT-R has two separate subscales: optimism and pessimism [37]. Thus, in the final analyses, we used the independent scores separately for optimism and pessimism.

After 3 years, in 2005, the study subjects were examined again. A total of 2625 subjects (93% of the original sample) had adequate responses in both 2002 and 2005, and could therefore be included in the final analyses. In 2005, the study subjects were asked if they had tried to improve or were about to improve their dietary habits, and if they had tried to improve their diet, how had they managed to achieve their goals. The possible improving styles in the diet were divided into five subgroups: reducing the consumption of fat, changing to low-fat products, reducing the consumption of sugar, increasing the consumption of vegetables, and increasing the consumption of berries and fruits.

We divided the study subjects in these five subgroups of different improving styles into four categories according to the possible changes in their diets: 1) those who had not tried to change their eating habits to a healthier diet, even when they thought it would have been beneficial, 2) those who thought their dietary habits were healthy enough even without an improvement, 3) those who had succeeded in improving of their diet, and 4) those who had tried to improve their diet but had failed to do so.

In the statistical analyses, we created dietary pattern models for grouping of the sample by using principal component analysis (PCA) with Varimax rotation and Kaiser normalization. Factor loadings with > 0.35 were considered as significant. Student’s t-test was used to study the associations between optimism, pessimism, and the different dietary patterns. When studying the differences in levels of optimism and pessimism, according to the success in the improvement of dietary habits in four categories, we used the Kruskal–Wallis test due to skewed distributions.

Finally, we calculated logistic regression models to discover the fully adjusted odds ratios for different variables for the risk of not succeeding in improving dietary habits.

## Results

Using the data from the food frequency questionnaire in 2002, we divided the study subjects into different dietary pattern groups by using principal component analysis. The analysis resulted in four nearly independent dietary patterns, which we named as ‘healthy’, ‘sweet unhealthy’, ‘fatty unhealthy’ and ‘traditional’ diets (Table 1). In further analyses, principal component analysis scores were used as independent variables to describe the amount of each different dietary pattern in the study subjects. We used the medians of the LOT-R optimism and pessimism subscale scores to classify the study subjects into low and high optimism and pessimism groups. Principal component analysis scores were compared between these groups (Table 2).

**Table 1:** Rotated factor matrix for dietary patterns created by using principal component analysis. Factor loadings with absolute values of > 0.35 have been presented in bold. Negative loadings indicate the lack of foodstuff in question belonging to certain dietary patterns

Foodstuff	Dietary pattern			
	Healthy	Sweet unhealthy	Fatty unhealthy	Traditional
Porridge, cereals	**0.382**	−0.001	−0.152	0.249
Fish	**0.397**	−0.109	0.060	−0.097
Lunch meats, cold cuts	**0.359**	0.214	0.055	0.142
Fresh vegetables/root vegetables	**0.664**	−0.018	−0.131	0.005
Cooked vegetables	**0.646**	−0.049	−0.032	− 0.098
Berries and fruits	**0.589**	0.076	−0.171	0.181
Fruit or berry juice	**0.378**	0.081	0.189	0.037
Sweet pastries	0.109	**0.597**	−0.031	0.256
Ice cream	0.088	**0.495**	0.085	−0.131
Candies	−0.043	**0.701**	0.033	0.078
Chocolate	0.035	**0.677**	0.098	−0.032
Salty snacks	−0.024	**0.352**	0.195	−0.221
Fried potatoes, French fries	−0.005	0.026	**0.489**	−0.059
Low-fat cheese	**0.411**	0.142	**−0.368**	−0.066
Other cheese	−0.004	0.025	**0.609**	0.108
Sausages	−0.147	0.240	**0.493**	0.065
Sliced sausages	−0.111	0.139	**0.558**	0.053
Eggs	0.151	0.013	**0.475**	−0.057
Soft drinks	−0.103	0.305	**0.352**	−0.125
Meat dishes	0.028	0.132	**0.366**	**0.552**
Chicken, turkey	**0.443**	0.003	−0.048	**−0.415**
Boiled or mashed potatoes	0.230	0.002	0.101	**0.658**
Rice, pasta	0.294	0.088	0.115	**−0.409**
Pizza, hamburgers	−0.021	0.263	0.169	**−**0.302

**Table 2:** Comparisons of principal component analysis scores of dietary patterns between groups with low or high pessimism, and low or high optimism

	Principal component analysis scores (mean)							
	Healthy dietary pattern	p ^1^	Sweet unhealthy dietary pattern	p ^1^	Fatty unhealthy dietary pattern	p ^1^	Traditional dietary pattern	p ^1^
Low pessimism (*N* = 1274) ^2^	0.071		0.029		−0.048		−0.006	
High pessimism (*N* = 1351) ^3^	−0.066	**< 0.001**	− 0.027	0.153	0.046	**0.016**	0.006	0.762
Low optimism (*N* = 1210) ^2^	−0.085		0.000		−0.019		0.026	
High optimism (*N* = 1415) ^3^	0.073	**< 0.001**	−0.000	0.995	0.016	0.365	−0.022	0.213

^1^ Student’s t-test; ^2^ Below the median; ^3^ Median or higher

p^1^-scores indicating statistical significance are bolded

At baseline, higher optimism and lower pessimism were associated with a ‘healthy’ dietary pattern. Optimism and pessimism did not seem to play any role in the ‘sweet unhealthy’ and ‘traditional’ dietary patterns, but high pessimism and the ‘fatty unhealthy’ dietary pattern associated significantly (Table 2).

The association between changes in dietary habits during the 3-year follow-up and pessimism was quite clear (Table 3). There was a strong trend that those who managed to change to a healthier diet were less pessimistic compared to others. The differences were statistically significant in four dietary categories: reducing fat, changing to low-fat products, increasing vegetables, and increasing berries and fruits. The higher the level of pessimism, the less likely was the improvement of diet. Nevertheless, those who had tried but failed reducing sugar were not more pessimistic than others. Optimism was associated with only one dietary change; those who had tried but failed to increase consumption of berries and fruits were less optimistic than others.

**Table 3:** The association between optimism and pessimism, and the change in dietary habits

	Has not changed	No need to change	Has changed	Tried to change, but failed	p^1^
Reducing fat	*N* = 82	*N* = 1059	*N* = 1280	*N* = 204	
Optimism (Mean (SD))	8.60 (2.02)	8.26 (2.24)	8.39 (2.08)	8.18 (2.14)	0.385
Pessimism (SD)	4.59 (2.60)	4.19 (2.79)	3.62 (2.58)	4.44 (2.81)	**< 0.001**
Changing to low-fat products	*N* = 155	*N* = 1098	*N* = 1266	*N* = 106	
Optimism (Mean (SD))	8.37 (2.20)	8.28 (2.21)	8.39 (2.09)	8.18 (2.15)	0.674
Pessimism (Mean (SD))	4.46 (2.74)	4.15 (2.77)	3.65 (2.60)	4.47 (2.76)	**< 0.001**
Increasing vegetables	*N* = 198	*N* = 1141	*N* = 1090	*N* = 196	
Optimism (Mean (SD))	8.46 (2.16)	8.25 (2.28)	8.43 (2.01)	8.10 (2.06)	0.058
Pessimism (Mean (SD))	4.10 (2.69)	4.09 (2.77)	3.69 (2.59)	4.43 (2.79)	**< 0.001**
Reducing sugar	*N* = 110	*N* = 1287	*N* = 986	*N* = 242	
Optimism (Mean (SD))	8.23 (2.13)	8.29 (2.23)	8.42 (2.04)	8.17 (2.18)	0.520
Pessimism (Mean (SD))	4.16 (2.54)	4.04 (2.75)	3.78 (2.69)	3.95 (2.54)	0.145
Increasing berries and fruits	*N* = 128	*N* = 1520	*N* = 859	*N* = 118	
Optimism (Mean (SD))	8.38 (2.05)	8.39 (2.20)	8.32 (2.05)	7.81 (2.22)	**0.041**
Pessimism (Mean (SD))	4.43 (2.77)	4.02 (2.78)	3.68 (2.51)	4.35 (2.72)	**0.002**

^1^ Kruskal–Wallis test

p^1^-scores indicating statistical significance are bolded

Finally, we calculated multivariate logistic regression models including several predicting variables for the risk of failure in improving dietary habits (Table 4). Because of the relatively small subgroups, we combined those who had failed in their dietary changes with those who had not even tried to improve their diet even when they recognized the need to do so into one group. We also combined those who saw no need to improve their diets with those who had managed to make healthy changes into another group.

**Table 4:** Odds ratios of different dietary pattern groups, coronary heart disease and pessimism (rows) on the risk of failure in change to more healthy dietary habits (columns) analysed by logistic regression models^a^

	Dietary change									
	No change and fail in reducing fat		No change and fail in changing to low-fat products		No change and fail in increasing vegetables		No change and fail in reducing sugar		No change and fail in increasing berries and fruits	
	OR	95% CI	OR	95% CI	OR	95% CI	OR	95% CI	OR	95% CI
Healthy dietary pattern	0.87	0.76–1.00	0.88	0.76–1.01	0.79	0.70–0.89	0.92	0.82–1.04	0.75	0.65–0.86
Sweet unhealthy dietary pattern	1.13	0.99–1.29	1.07	0.94–1.23	**1.26**	**1.13–1.40**	**1.30**	**1.16–1.45**	**1.23**	**1.08–1.40**
Fatty unhealthy dietary pattern	1.10	0.96–1.26	**1.14**	**1.00–1.31**	**1.17**	**1.05–1.32**	1.03	0.92–1.16	1.13	0.98–1.30
Traditional dietary pattern	1.12	0.98–1.27	1.02	0.90–1.17	0.97	0.87–1.08	1.01	0.90–1.14	0.89	0.78–1.02
Coronary heart disease	1.07	0.66–1.73	0.91	0.54–1.54	1.20	0.81–1.80	**1.52**	**1.00–2.31**	1.41	0.87–2.28
Pessimism	**1.07**	**1.02–1.12**	**1.07**	**1.02–1.13**	1.03	0.99–1.07	1.02	0.98–1.07	**1.05**	**1.00–1.11**

*OR* Odds ratio, *CI* Confidence interval

^a^Models are fully adjusted for age, sex, smoking and alcohol consumption habits, physical exercise, the levels of glucose, cholesterol and body mass index

p^1^-scores indicating statistical significance are bolded

The models included different dietary patterns, age, sex smoking and alcohol consumption habits, physical exercise, the levels of blood glucose and cholesterol, body mass index, the possible existence of CHD, and pessimism as explaining variables. A fatty unhealthy dietary pattern associated with the risk of failure in changing to low-fat products and in increasing vegetables. Sweet unhealthy dietary pattern associated with the risk of failure in increasing vegetables, in reducing sugar and in increasing berries and fruits. Finally, the effect of pessimism seemed clear in three out of five subgroups. Pessimism increased the probability of failure in reducing fat, changing to low-fat products, and increasing the consumption of berries and fruits.

To emphasize the association between pessimism and failures in changing dietary habits, we compared the highest and the lowest quarters of pessimism in logistic regression models which were fully adjusted for age, sex, smoking and alcohol consumption habits, physical exercise, the levels of glucose, cholesterol, body mass index and the possible existence of CHD. Those who belonged to the highest quarter of pessimism had a 1.4-fold risk of not succeeding in reducing their consumption of fat (adjusted OR 1.44, 95% CI 1.00–2.08, *p* = 0.05), a 1.5-fold risk of not succeeding in changing to low-fat products (adjusted OR 1.51, 95% CI 1.03–2.21, *p* = 0.03), and a 1.5-fold risk of failing to increase the consumption of berries and fruits in their diet (adjusted OR 1.46, 95% CI 1.01–2.12, *p* = 0.02) compared to the study subjects in the lowest quarter of pessimism.

## Discussion

Our main findings were that the dietary habits of study subjects with a higher level of pessimism were unhealthier compared to the dietary habits of others, and that the high level of pessimism was associated with greater difficulties in improving dietary habits. High levels of pessimism have been linked independently with an elevated risk of CHD [37–39]. While pessimism seems to be an independent risk factor for CHD, our results suggest that it may also be related to increased risk of CHD via an unhealthier diet.

There seemed to be no association between sweet unhealthy dietary pattern as well as fail in reducing sugar and optimism/pessimism. It has been speculated that the physiological and psychological mechanisms concerning sugar consumption might be different compared to the mechanism of other dietary habits. For example, when trying to eat healthily, the lack of sweet foods is often seen as the most difficult task [40] and when treating binge eating with baclofen, the medication seems to suppress binge eating of pure fat but not a sugar-rich diet [41].

It can be discussed whether the test subjects had proper information about good dietary habits, but it has been stated that the factor preventing people from eating healthy is not a lack of knowledge but rather the fact that people do not eat as healthily they know they should [1, 42, 43]. While there are many different recommendations about healthy diets which can make it challenging to know how to eat healthily it also seems that the correlation between nutrition knowledge and healthy dietary intake is quite weak [44].

Our study also strengthens the idea of optimism and pessimism as two different and independent variables. The statistical power of the optimism subscale was very small, while pessimism had stronger associations with several outcomes.

Improving the diet has a role in both prevention and treatment of several chronic diseases. The result of our study – pessimism being associated with difficulties in improving one’s diet – is parallel with earlier studies on psychosocial factors and adherence to various treatments. For example, adherence to treatment of asthma patients, hypertensive patients, cardiac patients, and rehabilitation patients after surgery seemed to relate to psychosocial factors, including dispositional optimism [45–48]. A higher level of optimism has also been associated, for example, with greater success in achieving good results in health changes among cardiac patients [49, 50] and in dental health [51]. Optimism and good compliance to treatment might also be connected in HIV patients [52].

An earlier study suggested that optimistic people exert greater efforts at goal attainment than pessimists do, for example, in alcoholism treatment [53]. In cross-sectional analyses, optimists have been shown to choose healthier foods when no preceding instructions are given [54, 55]. According to these studies, it seems that dispositional optimism and pessimism relate to the motivation in the treatment compliance, overall health behaviour, and the ability to make changes in lifestyle in order to improve physical well-being. The results of our study strengthen this claim.

As mentioned, there are some previous studies on associations between optimism/pessimism and dietary patterns [25–29]. However, there are some shortcomings in these studies. In these studies optimism and pessimism were dealt as a bipolar, single variable, and except for one study, the study participants were all of the same gender. It has been recognized in many other studies that optimism and pessimism are probably two independent variables that are present at same the time – i.e. one has both pessimistic and optimistic traits simultaneously [35]. The method of using optimism and pessimism as two different dimensions rather than one bipolar single variable may reveal much more information when the opposite ends of the bipolar variable do not cancel each other [32–36]. Separating optimism from pessimism turned out to be beneficial also in our study; optimism and pessimism seemed to be two different and independent factors as optimism seemed to have a connection with only one type of change in diet, while pessimism was associated much more strongly with many dietary behaviour changes. This endorses the need to separate optimism and pessimism to achieve more accurate results. Analysing optimism and pessimism as a unidimensional variable in this study would probably have covered some of the current results.

It has also been suggested that dispositional optimism might be a unidimensional continuum, but questions oriented pessimistically are better in determining this variable [54], thus diminishing the statistical power of optimistically oriented questions.

Even if it seems that people with high levels of pessimism have an unhealthier diet than others do and they are less likely to be able to change their dietary habits, it has been found that after proper education and monitoring, the association between pessimism and the ability to improve diet disappears. This conclusion was drawn following a trial derived from the GOAL study [56]. In the study, the subjects with higher pessimism levels had unhealthier lifestyles, including unhealthier dietary habits. However, after the pessimists had received education concerning healthier lifestyles and were subjected to close monitoring, they managed to improve their lifestyles equally to other subjects. Keeping this in mind, it would seem only natural that determining pessimism could help in finding those who probably have unhealthier diets and are in greater risk in failing to improve them. Those subjects could then be targeted with proper education about healthy diets, and the monitoring of dietary changes could lower the risk of various diseases. Naturally, the independent risk of pessimism in developing those illnesses – for example, CHD – is still unlikely to diminish. Determining the level of dispositional pessimism is quick to assess and practically cost free, so it can be expected to be very cost-effective.

There are some strengths and weaknesses in our study and methods. The population was drawn as a random sample and it is representative of Lahti Region with 200,000 inhabitants. However, it seems that poorly functioning and institutionalized persons had a lower participation rate than community-dwelling subjects [57]. The design is longitudinal and observational, but it can obviously not detect any causality between the assessed variables. We have measured a great number of variables, hence the possibility to adjust for a number of confounders was good. However, and typical of cohort studies, the methods were mostly simple and we were unable to describe the diet by, e.g., an extensive food-frequency questionnaire. In the analyses, we classified reduction of fat as an indication of a healthy change. This may of course be debated, since more recent studies indicate that fat quality (shift from saturated towards unsaturated fats) is more important than the intake of total fat per se [58]. In early 2000’s, reductions in dietary fat and in fatty foods were generally - at least among many lay individuals - regarded as healthy. Hence, we chose to use fat reduction as an indication of a choice to improve dietary quality.

Much of the data used in this study is based on self-rated questionnaires, so there might be some inconsistency between the answers and the reality in the questions concerning, for example, smoking habits and use of alcohol.

## Conclusions

Dietary habits play an important role in the development of many diseases, and improving the diet reduces the risk for developing many severe illnesses. Pessimism and to some extent optimism seem to play a role in current dietary habits and in the ability to change these habits. By determining optimism and particularly pessimism, it is possible to detect individuals in greater need of guidance and support in ameliorating their dietary habits. Separating optimism and pessimism seems to make a clearer connection between the optimism construct and dietary habits as well as between the optimism construct and the ability to make healthy dietary changes.

## Abbreviations

BMI: Body mass index
CHD: Coronary heart disease
FFQ: Food frequency questionnaire
GOAL: Good Ageing in Lahti Region study
LOT: Life orientation test
LOT-R: Revised version of the life orientation test
PCA: Principal component analysis
